# Biglycan Regulates MG63 Osteosarcoma Cell Growth Through a LPR6/β-Catenin/IGFR-IR Signaling Axis

**DOI:** 10.3389/fonc.2018.00470

**Published:** 2018-10-23

**Authors:** John Aggelidakis, Aikaterini Berdiaki, Dragana Nikitovic, Antonis Papoutsidakis, Dionysios J. Papachristou, Aristidis M. Tsatsakis, George N. Tzanakakis

**Affiliations:** ^1^Laboratory of Anatomy-Histology-Embryology, School of Medicine, University of Crete, Heraklion, Greece; ^2^Unit of Bone and Soft Tissue Studies, Laboratory of Anatomy-Histology-Embryology, School of Medicine, University of Patras, Patras, Greece; ^3^Laboratory of Toxicology, School of Medicine, University of Crete, Heraklion, Greece

**Keywords:** osteosarcoma growth, biglycan, small leucine rich proteoglycans, IGF-IR, β-catenin, extracellular matrix

## Abstract

Biglycan, a small leucine rich proteoglycan (SLRP), is an important participant in bone homeostasis and development as well as in bone pathology. In the present study biglycan was identified as a positive regulator of MG63 osteosarcoma cell growth (*p* ≤ 0.001). IGF-I was shown to increase biglycan expression (*p* ≤ 0.01), whereas biglycan-deficiency attenuated significantly both basal and IGF-I induced cell proliferation of MG63 cells *(p* ≤ 0.001; *p* ≤ 0.01, respectively). These effects were executed through the IGF-IR receptor whose activation was strongly attenuated (*p* ≤ 0.01) in biglycan-deficient MG63 cells. Biglycan, previously shown to regulate Wnt/β-catenin pathway, was demonstrated to induce a significant increase in β-catenin protein expression evident at cytoplasmic (*p* ≤ 0.01), membrane (*p* ≤ 0.01), and nucleus fractions in MG63 cells (*p* ≤ 0.05). As demonstrated by immunofluorescence, increase in β-catenin expression is attributed to co-localization of biglycan with the Wnt co-receptor low-density lipoprotein receptor-related protein 6 (LRP6) resulting in attenuated β-catenin degradation. Furthermore, applying anti-β-catenin and anti-pIGF-IR antibodies to MG-63 cells demonstrated a cytoplasmic and to the membrane interaction between these molecules that increased upon exogenous biglycan treatment. In parallel, the downregulation of biglycan significantly inhibited both basal and IGF-I-dependent ERK1/2 activation, (*p* ≤ 0.001). In summary, we report a novel mechanism where biglycan through a LRP6/β-catenin/IGF-IR signaling axis enhances osteosarcoma cell growth.

## Introduction

The small leucine-rich proteoglycans (SLRPs) are a family of proteoglycans (PGs) characterized by a relatively small protein core (36–42 kDa), containing a number of leucine-rich repeats and able to undergo post-translational modifications, notably substitution with various types of glycosaminoglycan side chains (1, 2). The PGs secreted to the extracellular matrix can be found at multiple locations, have a high level of conservation and are expressed at crucial points in embryogenesis and tissue homeostasis, which is indicative of their importance. The SLRP gene family now counts 18 genes classified into five distinct subfamilies which is based on their chromosomal organization and functional similarities (3).

Biglycan is a canonical class I SLRP member containing two chondroitin or dermatan sulfate side chains which are covalently bound to attachment sites at the N-terminal (4). Recently, novel roles in cancer biology including regulation of proliferation, apoptosis, migration, motility, inflammation as well as autophagy have been attributed to biglycan (5, 6). The effects of biglycan have likewise been implicated in the pathogenesis of osteosarcoma, primary malignant tumor of the bone (7, 8). The highest incidence of this tumor has been reported in children and young adults between the ages of 10 and 30 (9, 10) with a significant 30–40% of patients still exhibiting relapses and adverse outcome despite major advances in the treatment of this cancer (10, 11). An important characteristic of osteosarcoma is its heterogeneity and ability to produce abundant non-mineralized ECM–osteoid, mainly consisting of collagen type I, glycoproteins, and proteoglycans (PGs)/GAGs (12, 13). Specifically, biglycan has been identified as a product involved in resistance to chemotherapy-resistant pediatric osteosarcoma (14). Furthermore, we have previously shown that parathyroid hormone (PTH) fragment, PTH(1-34), and fibroblast growth factor-2 (FGF-2), through a novel cooperative mechanism of action, modulate the extracellular matrix content of biglycan to regulate osteosarcoma cell migration (15, 16). Key downstream mediators of biglycan have been correlated to osteosarcoma development and prognosis. Thus, it was recently demonstrated that the attenuation of the low-density lipoprotein receptor-related protein 6 (LRP6)-Wnt/β-catenin signaling pathway through microRNA-183 (miR-183) inhibits osteosarcoma cell proliferation and invasion (17). Wnt pathway is suggested as one of key pathways in osteosarcoma pathogenesis (18). Moreover, the Wnt/β-catenin pathway is a therapeutic target in osteosarcoma as its suppression through resveratrol action elicits anti-tumor effects (19). Other SLRP have likewise been shown to affect various osteosarcoma cell functions including proliferation, migration or adhesion (20–22). In the present work, taking into account the importance of biglycan and its downstream mediators in the functional regulation of osteoblastic lineage cells, we examined the effect of biglycan on osteosarcoma cell growth as well as the putative mechanisms involved. Our findings revealed that biglycan through an LPR6/β-catenin/IGF-IR signaling axis positively regulates MG63 osteosarcoma cell growth.

## Materials and methods

### Materials

Recombinant human biglycan (2667-CM) and IGF-I (insulin-like growth factor I; 291-G1) were obtained from R&D Diagnostics. Selective inhibitor of ERK1/2 (Cell Signaling Technology; U016) and allosteric inhibitor of IGF-IR (Sigma-Aldrich; AG1024) were used in this study. Primary antibodies from Santa Cruz Biotechnology used, anti-biglycan (sc100857; mouse monoclonal; 1/100 dilution), anti-β catenin (sc7963; mouse monoclonal; 1/300 dilution and 1/50 for immunofluorescence experiments), anti-IGF-IR total (sc81464; mouse monoclonal; 1/100 dilution), anti-pERK1/2 (sc136521; mouse monoclonal; 1/100 dilution) and anti-fibrillarin (sc374022; 1/100 dilution). In addition, anti-actin (Millipore MAB1501; mouse monoclonal; $\frac{1}{2}$,500 dilution), anti-ERK total (Thermo scientific MA5-15343; mouse monoclonal; 1/500 dilution), anti-pIGF-IR (Thermo scientific PA5-37602; polyclonal rabbit; 1/500 dilution for western blot and 1/50 for immunofluorescence experiments), anti-tubulin (Sigma-Aldrich T4026; monoclonal mouse; 1/1,000 dilution) and anti-LRP6 (Elabscience E-AB17347; rabbit polyclonal; 1/50 dilution) were utilized. Secondary-HRP antibodies anti-rabbit (AP182PR); and anti-mouse (AP192PM) were used in a 1/3,000 dilution and obtained from Millipore.

### Cell culture

In this study, MG63 **(ATCC® CRL1427™)** human osteosarcoma cell line of moderately differentiated fibroblastoid-type cells and high metastatic capacity was utilized. Cells were grown in DMEM (Biochrom AG; F0455) supplemented with 10% fetal bovine serum (FBS; Invitrogen 10500-064; heat inactivated), glutamine (4 mM; Biosera XCT1715), gentamycin (Invitrogen; 15710-049) and penicillin/streptomycin (100 units/ml; Biosera LMA4118). Before addition of treatments (biglycan 10 μg/ml or IGF-I 10 ng/ml), cells were cultured in serum free medium for 24 h at 37°C and 5%CO_2_. Inhibitors, when used (ERK inhibitor or IGF-IR inhibitor), were added 1 h before growth factor treatment.

### Proliferation assay

Growing cells from non-confluent cultures were harvested and seeded in black 96-well plates (Corning; 3603) at a density of 3,500 cells per well in 200 μl of DMEM (10% FBS). The cell density number was chosen from optimization experiments (data not shown). The cells were allowed to rest overnight. If necessary, transfection with siRNAs was performed in a serum-free medium without antibiotics for 6 h. This was then replaced with fresh medium (0% FBS) with antibiotics. Treatments were added for the next 48 h at 37°C and 5% CO_2_ in 0% FBS. The cells were then lysed and their number was calculated using the CyQUANT fluorometric assay (Thermo Scientific; C7026) according to the manufacturer's instructions. Fluorescence was measured in a Fluorometer (Biotek) using the proposed excitation (485 nm) and emission filters (528 nm). A separate standard curve was used to convert fluorescence units to cell numbers. All experiments were performed in triplicate.

### Transfection with siRNA

For transfection experiments, the cells were plated in serum and antibiotic free medium in either 96 well plates (4,000 cells/well), or 24-well plates (80,000 cells/well) or T25 flasks (1:8 dilution of a 90% confluent T75 flask). Short interfering RNA specific for biglycan (siBGN) [Invitrogen; stealth siRNAs HSS184531, S328618; optimized for MG63 cells by (15)] or β-catenin (sib-cat) (sc29209; Santa Cruz Biotechnology), and RNAi negative control (siScr) (Invitrogen; medium GC content negative control). To provide optimal transfection, siRNA and Lipofectamine 2000 (Invitrogen; 11668-027) were diluted in Opti- MEM I Reduced Serum Medium (Invitrogen; 31985-070). After 5 min of incubation, diluted Lipofectamine 2000 was mixed with diluted siRNA (2 μM) for 20 min at room temperature to allow siRNA–liposome complexes to form and added to cell layers. Transfection was allowed to take place during 6 h when the medium was replaced with fresh serum-free medium containing antibiotics and the incubation period continued for 48 h. Cells were then harvested and mRNA or protein was extracted. When necessary, treatments were performed during 24 h after the initial 48 h transfection period. All transfection experiments were repeated at least three times and performed in triplicates.

### RNA isolation and real-time PCR

Total ribonucleic acid isolation was performed using TRIzol (Invitrogen; 15596026), according to the manufacturer's instructions. One microgram of total RNA was added for cDNA synthesis using the TAKARA (RR037A) RT cDNA synthesis kit. For semi-quantification of the genes of interest, real-time reactions were performed in an Mx300P cycler using the Universal qPCR kit (KAPA Biosystems; KK4602) in a total volume of 20 μL. PCR conditions for amplification and primers used are listed in Table 1. Standard curves were run in each optimized assay, which produced a linear plot of threshold cycle (Ct) against log (dilution). The amount of each target was quantified based on the concentration of the standard curve and was presented as arbitrary units. GAPDH was utilized as a housekeeping control gene

**Table 1:** Real-time PCR primers and amplification conditions.

**Primers**	**Sequences**
GAPDH	Forward: 5′-GGA AGG TGA AGG TCG GAG TCA-3′ Reverse: 5′-GTC ATT GAT GGC AAC AAT ATC CAC T-3′
β-catenin	Forward: 5′-TTC TGG TGC CAC TAC CAC AGC-3′ Reverse: 5′-TGC ATG CCC TCA TCT AAT GTC-3′
Cyclin	Forward: 5′-CTC CAC CTC ACC CCC TAA AT-3′ Reverse: 5′-AGA GCC CAA AAG CCA TCC-3′
Biglycan	Forward: 5′-TCT GAA GTC TGT GCC CAA-3′ Reverse: 5′-TCT GAG ATG CGC AGG TA-3′
Thermal conditions	94°C for 15 min; 40 cycles at 94°C for 20 s; 55°C for30 s; 72°C for 30 s; 72°C for 10 min

### Western blot

Total protein secreted into the serum-free culture medium was concentrated using Amicon Ultra 15 mL (UFC901024; 10 kDa cutoff) centrifugal concentrator tubes. The initial volume of 3 mL serum-free medium collected from culture, to isolate secreted proteins, was concentrated to final volume of 500 μL whereas, harvested cells were lysed with RIPA solution (50 mM Tris-HCl, 1% NP-40, 0.25% Na-Deoxycholate, 150 mM NaCl, 1 mM EDTA with protease and phosphatase inhibitors). Equal amounts of protein, either cell extracts or secreted, were subjected to SDS-PAGE using 8% polyacrylamide gels under reducing conditions. Separated protein bands were transferred to nitrocellulose membranes in 10 mM (pH 11), containing 10% methanol. Membranes were blocked overnight at 4°C with PBS containing 0.1% Tween-20 (PBS-Tween) and 5% (w/v) low-fat milk powder. The membranes were incubated for 1 h at room temperature (RT) with primary antibody in PBS containing 0.1% Tween-20 (PBS-Tween) and 1% (w/v) low-fat milk powder. The immune complexes were detected after incubation with the appropriate peroxidase-conjugated secondary antibody diluted (1:3,000) in PBS-Tween, 2% low-fat milk, using the LumiSensor Chemiluminescent HRP substrate kit (Genscript; L00221V500), according to the manufacturer's instructions. Protein expression of Actin was used to correct for the amount of each sample analyzed.

### Nuclear and cytoplasmic extract separation

Treated cells in T25 flasks were detached using trypsin-EDTA (Biosera; LMT1706), deactivated with PBS and centrifuged at 1,100 rpm for 5 min. Supernatants were discarded and the pellets resuspended in 250 μL of ice cold PBS supplemented with protease and phosphatase inhibitors. After centrifugation at 1,100 rpm for 5 min at 4°C the pellets were resuspended in 200 μL of 5x CPV NP-40 lysis buffer (10 mM Tris-HCl, 10 mM NaCl, 3 mM MgCl_2_, 0.5% NP-40 with protease and phosphatase inhibitors). Tubes were incubated on a rotating platform at 4°C for 10 min and centrifuged strictly at 1,000 rpm for 5 min at 4°C. The supernatants (cytoplasmic protein fractions) were kept at −80°C. The pellets were resuspended in 100 μL of RIPA solution (50 mM Tris-HCl, 1% NP-40, 0.25% Na-Deoxycholate, 150 mM NaCl, 1 mM EDTA with protease and phosphatase inhibitors), vortexed and incubated for 60 min on a rotator at 4°C. Samples were centrifuged to pellet insoluble fraction at 13,000 rpm for 30 min at 4°C and after the supernatants (nucleus protein fractions) were kept at −80°C as well as the pellets (membrane fractions).

### Immunofluorescence

MG63 cells were seeded on round coverslips placed in 24-well plates, at a concentration of 50,000 cells/well and incubated in complete medium for 24 h. After a 24-h serum starvation, treatments were added and the cells incubated for 48 h at 37°C and 5% CO_2_. The cells were fixed with 5% formaldehyde and 2%sucrose in PBS for 10 min at RT. After three washes with PBS supplemented with 1% FBS, the permeabilizing agent Triton X-100 was added for 10 min and then washed before the addition of primary antibody for 1 h at RT. Coverslips not incubated with the primary antibody were utilized as negative controls. The coverslips were washed again and incubated for 1 h, in the dark at RT, with anti-mouse Alexa 555 (Thermo Scientific; A21422) or Alexa Fluor 488 (Molecular Probes; A21206). TO-PRO-3 iodode (Molecular Probes; T3605) diluted 1:1,000 in de-ionized H_2_O was applied for 10 min to stain nuclei. The coverslips were then placed onto slides using glycerol as a mountant and visualized using confocal microscopy.

### Statistical analysis

The statistical significance was evaluated by student's *t*-test, or ANOVA analysis of variance with Tukey's post-test, using GraphPad Prism (version 4.0) software.

## Results

### The role of biglycan on IGF-I stimulated MG63 cell proliferation

**Figure 1:**
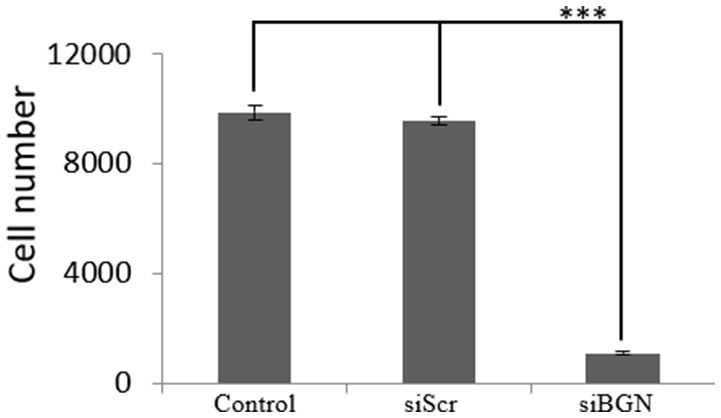
Effect of siBGN on MG63 cell proliferation. MG63 cells were harvested and seeded (3,500 cells/well) on 96-well plates and transfection with siRNAs (short interfering RNAs) was performed. Cells, in each well, were incubated in serum-free medium and transfected with either siRNAs against biglycan (siBGN) or scrambled siRNAs (siScr), used as negative control. Cells were counted after a 48 h incubation period, using fluorometric CyQUANT assay kit. Results represent the average of three separate experiments. Means ± S.E.M were plotted; statistical significance: ^***^p ≤ 0.001 compared with the respective control samples.

We have previously shown that biglycan is a potent modulator of osteosarcoma cells' migration (15). In this study we wanted to investigate its' possible role on osteosarcoma cell growth. Therefore, we used siRNAs specific for the biglycan gene (siBGN) achieving a significant downregulation of biglycan expression at both protein and mRNA biglycan levels, as previously shown (15). The growth ability of biglycan-deficient and control siScr cells was evaluated using the CyQUANT fluorometric assay (20). This approach demonstrated a strong attenuation of biglycan deficient cell growth as compared to control (*p* ≤ 0.001; Figure 1).

### IGF-I modulation of biglycan expression

**Figure 2:**
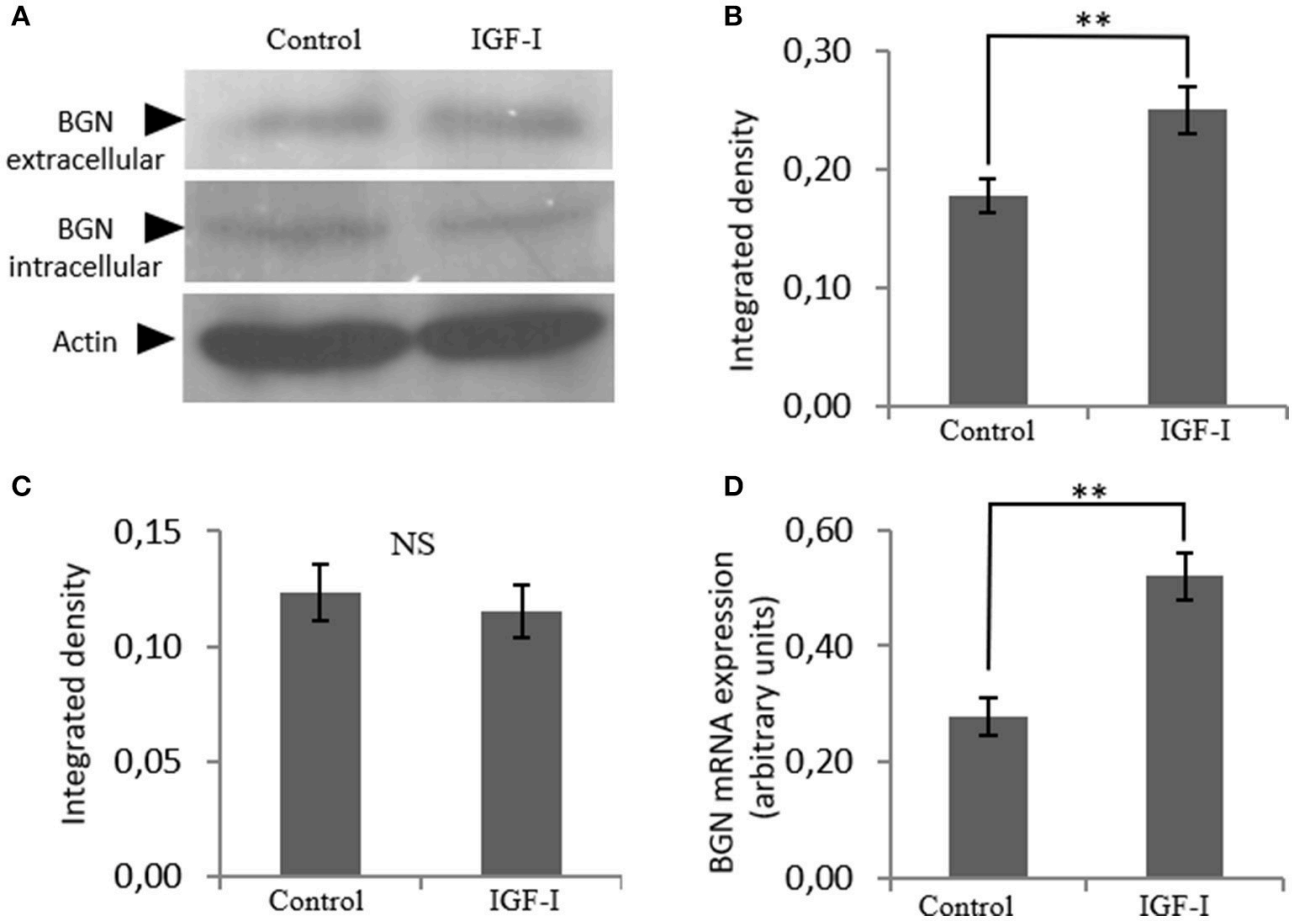
Effect of IGF-I on biglycan expression at the mRNA and protein level. **(A)** Expression of extracellular and intracellular Biglycan (BGN) levels of cells treated with serum-free medium (control) and cells treated with IGF-I (10 ng/ml) was determined by Western blot analysis. Densitometric analysis of the extracellular BGN protein band (100 KDa glycosylated proteoglycan) **(B)** and of the intracellular BGN protein band (45 KDa protein core band) **(C)** were normalized against actin and plotted. Representative blots are presented. **(D)** Biglycan mRNA levels in MG63 cells treated with IGF-I (10 ng/ml) during 48 h were determined by real time PCR using primers specific for the BGN gene and normalized against GAPDH. Results represent the average of three separate experiments. Means ± S.E.M were plotted; statistical significance: ^**^p ≤ 0.01 compared with the respective control samples.

In order to identify possible partners/mediators of biglycan action we screened the effect of key regulators of osteosarcoma growth on biglycan expression. This approach identified IGF-I as a regulator of biglycan expression. Indeed, upon treating MG63 with IGF-I (10 ng/mL) for 48 h and performing western blot analysis to supernatant and cell extract, a statistically significant increase of secreted biglycan (*p* ≤ 0.01), was demonstrated (Figure 2). Utilization of antibody specific for actin on secreted proteins excluded a contamination by cytoskeletal proteins (data not shown). Biglycan mRNA levels were also significantly (*p* ≤ 0.01) upregulated, as shown by real-time PCR analysis (Figure 2D). These data are well in accord with previous reports where IGF-I has been shown to regulate the expression of biglycan in human osteoblast-like cells (23).

**Figure 3:**
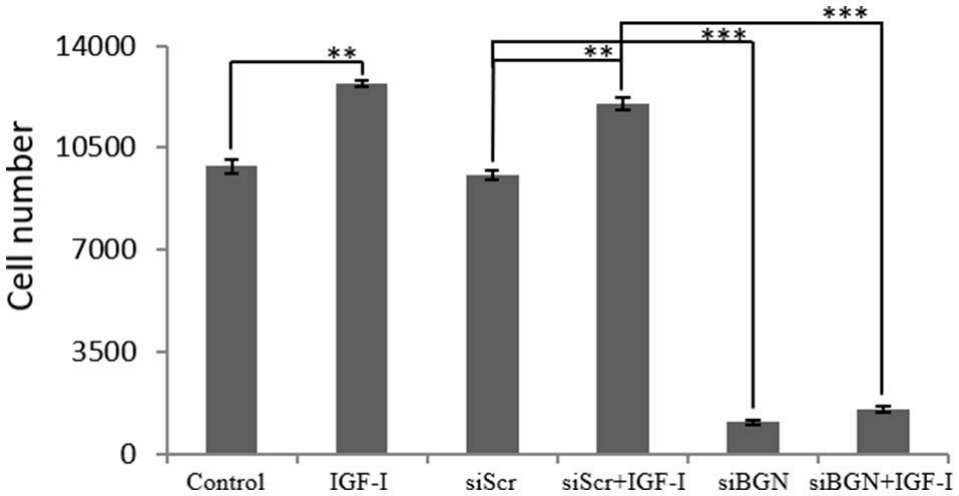
Effect of IGF-I on cell proliferation of MG63 cells. MG63 cells were harvested and seeded (3,500 cells/well) on 96-well plates and transfection with siRNAs was performed. Cells, in each well, incubated with 0% FBS-medium (control), cells incubated with 10 ng/ml IGF-I (IGF-I) and cells transfected with either siRNAs against biglycan (siBGN) or scrambled siRNAs (siScr) with or without IGF-I addition, were counted using fluorometric CyQUANT assay kit. Results represent the average of three separate experiments. Means ± S.E.M were plotted; statistical significance: ^***^p ≤ 0.001, ^**^p ≤ 0.01 compared with the respectivecontrol samples.

Due to the fact that, IGF-I/IGF-IR is a key signaling pathway of bone anabolic processes and established in early reports to regulate osteosarcoma cell proliferation (24) we wanted to verify its putative action on MG63 cell growth and assess possible connection to biglycan effects. Treating osteosarcoma cells with IGF-I (10 ng/ml) induced a significant increase in cell proliferation (*p* ≤ 0.01; Figure 3). To estimate an interaction between biglycan and IGF-I signaling we treated biglycan-deficient cells (siBGN) as well as cells transfected with control scramble siRNAs (siScr) with IGF-I (10 ng/mL) for 48 h and measured their proliferation rate. IGF-I-induced increase in cell proliferation (*p* ≤ 0.01) was abolished in biglycan-deficient cells (*p* ≤ 0.001; Figure 3). Therefore, biglycan was shown to modulate significantly both basal and IGF-I induced cell proliferation of MG63 cells, suggesting an interplay between biglycan and IGF-I signaling in the regulation of osteosarcoma growth.

### Role of IGF-IR on IGF-I-dependent MG63 cell proliferation—effect of biglycan

**Figure 4:**
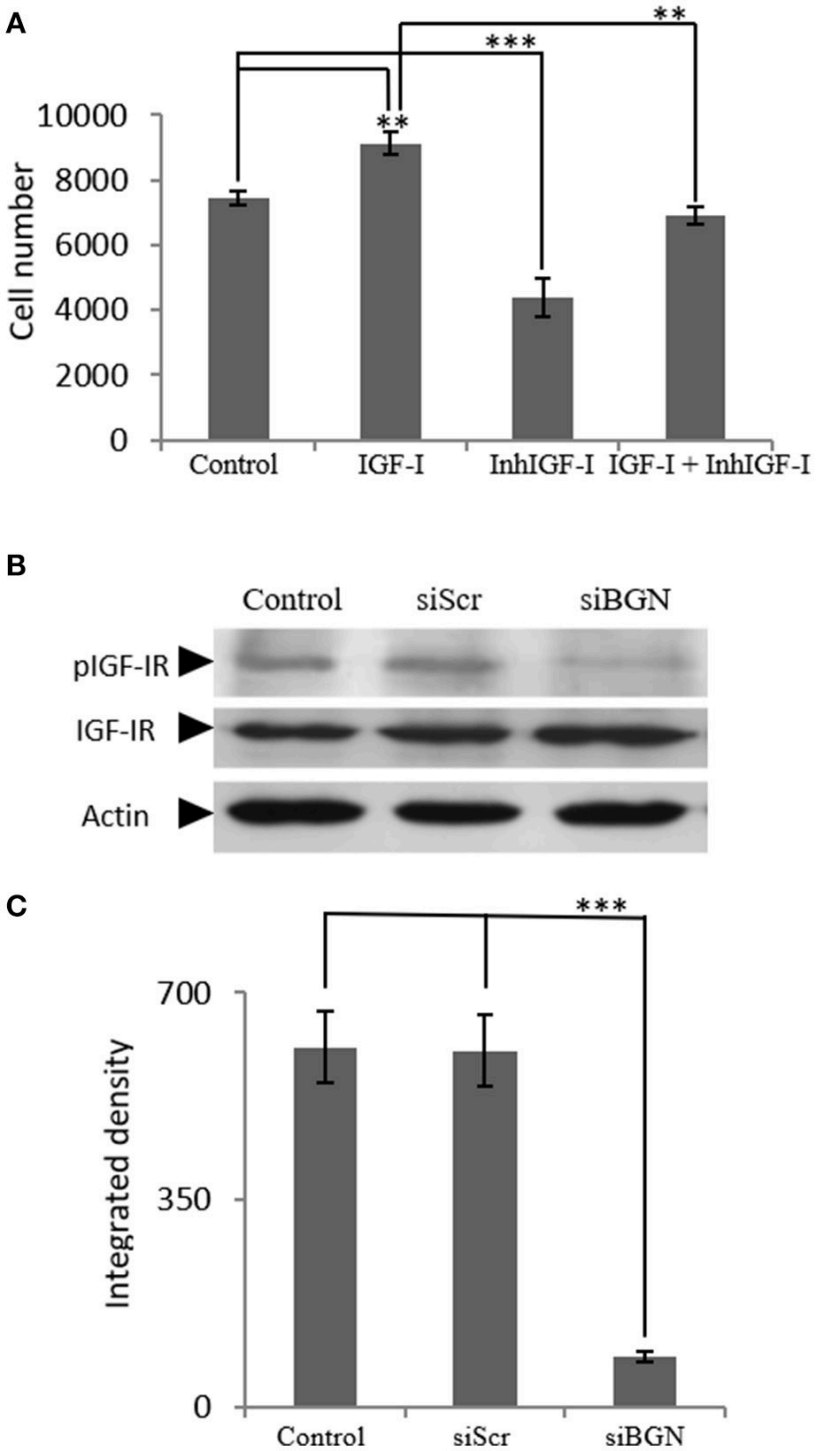
Role of IGF-IR on IGF-I-dependent MG63 cell proliferation- effect of biglycan. **(A)** Effect of IGF-Inhibitor on cell proliferation of MG63 cells. MG63 cells were harvested and seeded (3,500 cells/well) on 96-well plates. Cells, in each well, incubated with 0% FBS-medium (control), 10 ng/ml IGF-I (IGF-I), 1 μM IGF-I inhibitor (InhIGF-I), and 10 ng/ml IGF-I + 1 μM IGF-I inhibitor (IGF-I+ InhIGF-I), were counted using fluorometric CyQUANT assay kit. **(B)** Effect of siBGN on the activation of IGF-IR. Expression of IGF-IR total protein (IGF-IR) and phosphorylated IGF-IR protein (pIGF-IR) of cells in serum-free medium (control) and cells transfected with either siRNAs (short interfering RNAs) against biglycan (siBGN) or scrambled siRNAs (siScr) were determined by Western blot analysis. **(C)** Densitometric analysis of the activated IGF-IR levels (pIGF-IR/IGF-IR) from the protein and were normalized against actin and plotted. Representative blots are presented. Results represent the average of three separate experiments. Means ± S.E.M were plotted; statistical significance: ^***^p ≤ 0.001, ^**^p ≤ 0.01 compared with the respective control samples.

Next, we examined the mechanisms involved in IGF-I-dependent growth, taking into account the fact that the IGF-IR receptor is the key IGF-I downstream mediator (25) as well as the confirmation of IFG-IR activation shown in our control experiments (Supplementary Figure 1). For this purpose MG63 cells were treated with 1 μM of specific IGF-IR inhibitor (AG1024) for 48 h, with or without the presence of IGF-I (10 ng/ml). The AG1024 concentration used was chosen after initial optimization experiments that were performed using a range of different concentrations (data not shown). Fluorometric cell growth assay demonstrated that this strategy resulted in a statistically significant decrease of both basal and IGF-I-induced proliferation (*p* ≤ 0.001; *p* ≤ 0.01, respectively; Figure 4A). These results, therefore, suggest that IGF-IR mediates both basal and exogenous IGF-I-dependent growth of MG63 osteosarcoma cells. Of note, these data highlight the importance of endogenous IGF-IR signaling in the regulation of MG-63 cell growth (Figure 4A).

In order to investigate the direct effects of biglycan on IGF-IR downstream signaling, the activation of IGF-IR was studied. To this end, cell extracts of siBGN and control siScr cells, were probed with antibodies against IGF-IR and pIGF-IR. As presented in Figures 4B,C, the activation of IGF-IR was strongly attenuated (*p* ≤ 0.01) in biglycan-deficient MG63 cells (Figures 4B,C).

### Biglycan mediated changes in the Wnt pathway (β-catenin)

**Figure 5:**
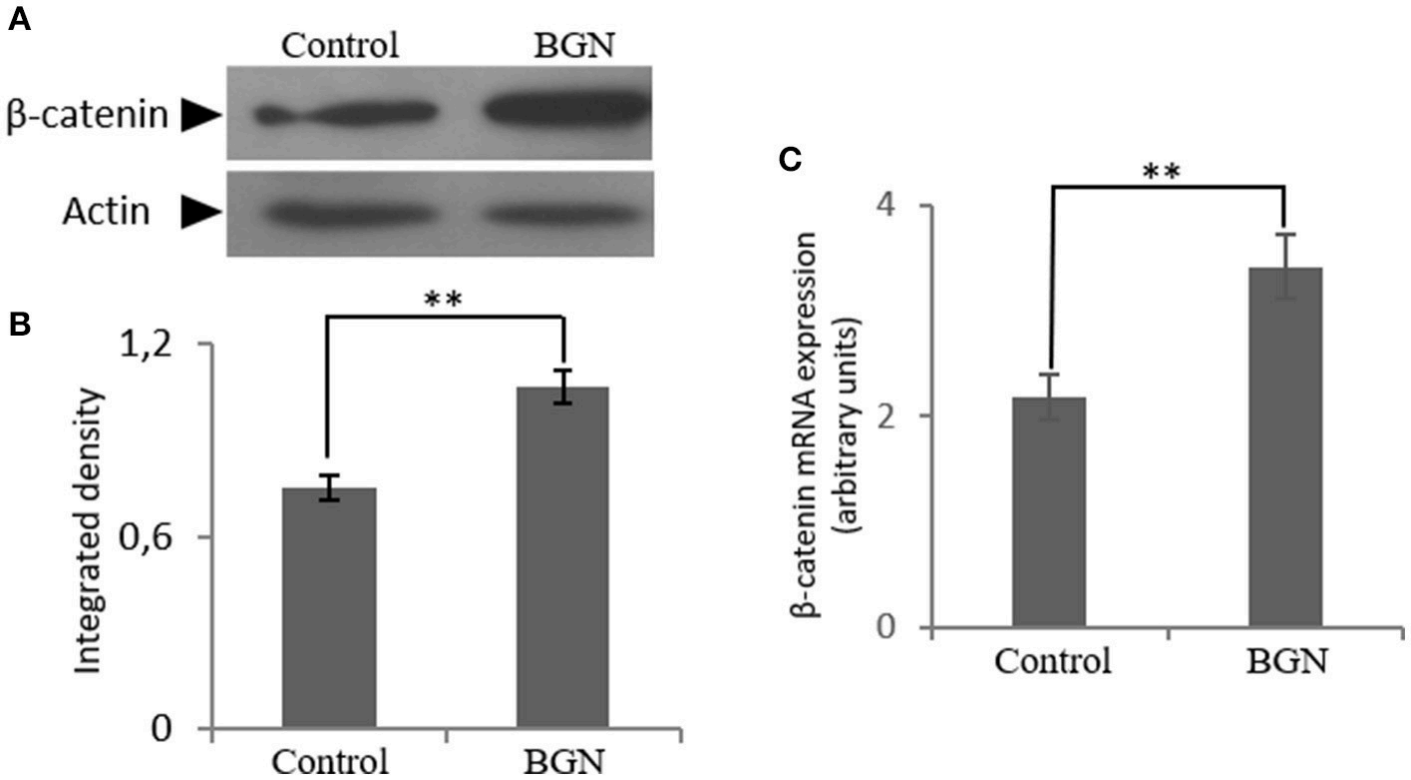
Effect of biglycan on the expression of β-catenin at the mRNA and protein level. **(A)** Expression of β-catenin in cells treated with 0% FBS-medium (control) and cells treated with biglycan (BGN; 10 μg/ml) was determined by Western blot analysis. **(B)** Densitometric analysis of the β-catenin protein bands were normalized against actin and plotted. Representative blots are presented. **(C)** B-catenin mRNA levels in MG63 cells treated with biglycan (BGN) during 48 h were determined by real time PCR using primers specific for the β-catenin gene and normalized against GAPDH. Results represent the average of three separate experiments. Means ± S.E.M were plotted; statistical significance: ^**^p ≤ 0.01 compared with the respective control samples.

Next, to further define the mechanism of biglycan action, we turned to the identification of putative co-operation partners. Biglycan has been suggested to regulate Wnt/β-catenin pathway by either mediating ligand effects or modulating downstream signaling molecules (26–29). Indirect interactions of β-catenin with IGF-IR receptor and other members of the IGF-I signaling pathway have also been reported (27–29). In order to investigate the effect of biglycan on the Wnt pathway in osteosarcoma cells, β-catenin expression was studied after incubation of the cells with biglycan for 48 h. Both protein (Western blot) and mRNA (Real time PCR) analysis showed a statistically significant increase (*p* ≤ 0.01) in β-catenin protein expression after the addition of biglycan (Figure 5). This increase in β-catenin protein expression is well in correlation with a previous report in normal human osteoblasts (26).

**Figure 6:**
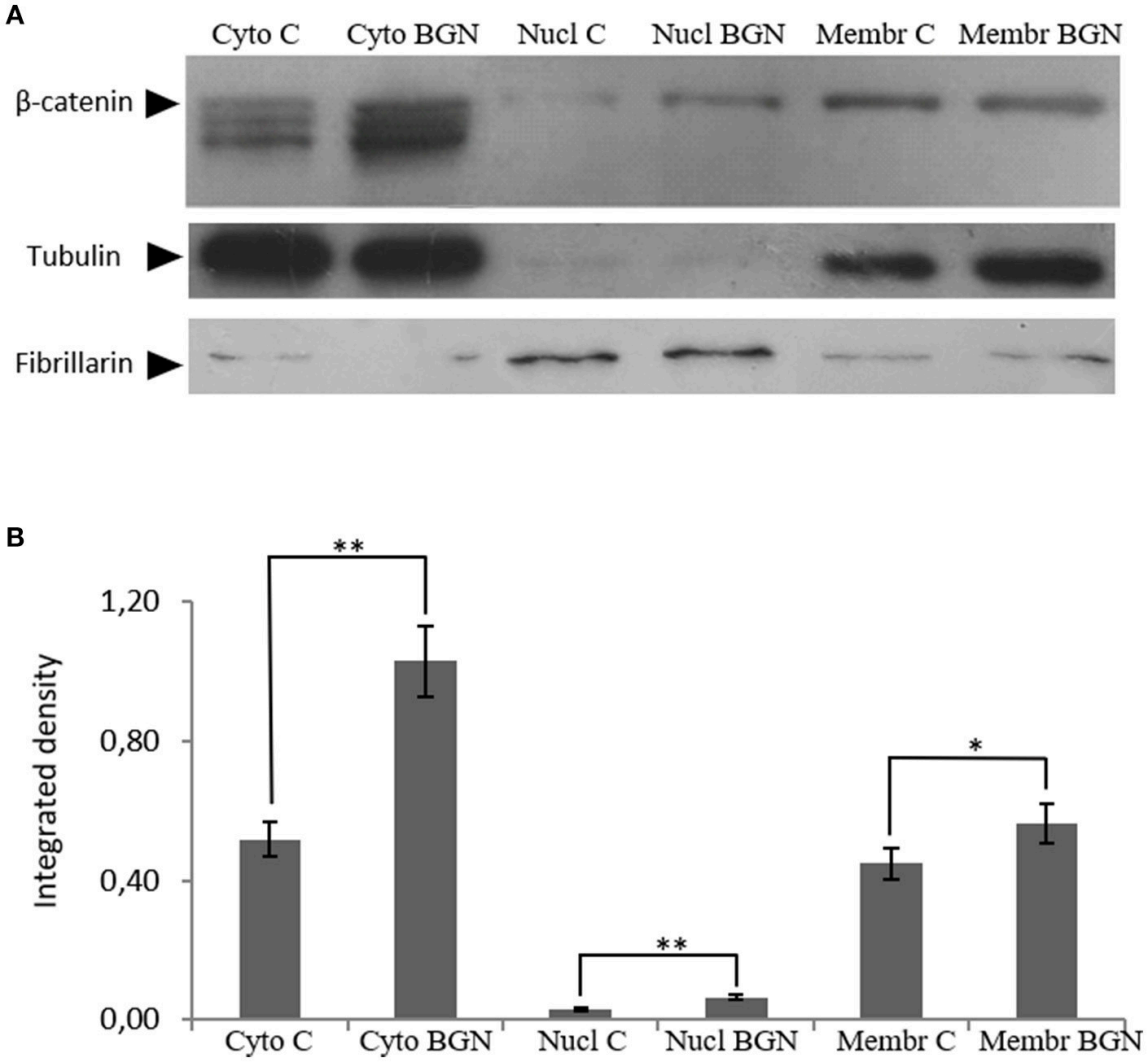
Effect of biglycan on β-catenin protein expression at the different MG63 cell compartments. **(A)** Expression of β-catenin in the cytoplasmic compartment of the cells treated with 0% FBS-medium (Cyto C) and cells treated with biglycan 10 μg/ml (Cyto BGN), as well as the nuclear compartment of the cells (Nucl C; Nucl BGN) and the membranes (Membr C; Menbr BGN) was determined by Western blot analysis. **(B)** Purity controls tubulin and fibrillarin were used for cytoplasmic and nuclear proteins, respectively. Equal amounts of protein from each compartment were loaded and densitometric analysis was performed and plotted. Representative blots are presented. Results represent the average of three separate experiments. Means ± S.E.M were plotted; statistical significance: ^**^p ≤ 0.01, ^*^p ≤ 0.05 compared with the respective control samples.

β-catenin is known to regulate gene expression following Wnt pathway activation which affects cell proliferation (30–32). The above functions of β-catenin derive from its interplay with several proteins in different compartments e.g., the cell membrane, the cytoplasm and the nucleus. Therefore, β-catenin localization was investigated at the different compartments of MG63 cells after treatment with biglycan during 48 h. As presented in Figure 6 a significant increase in the expression of β-catenin to the cytoplasm (Cyto C vs. CytoB; *p* ≤ 0.01); to the nucleus (NuclC vs. NuclB; *p* ≤ 0.01) as well as to the membrane (MembrC vs. MembrB; *p* ≤ 0.05) was demonstrated in biglycan treated cells as compared to control. Therefore, biglycan regulates the protein expression and localization of β-catenin in MG63 osteosarcoma cells.

### Role of β-catenin in osteosarcoma IGF-1R signaling

**Figure 7:**
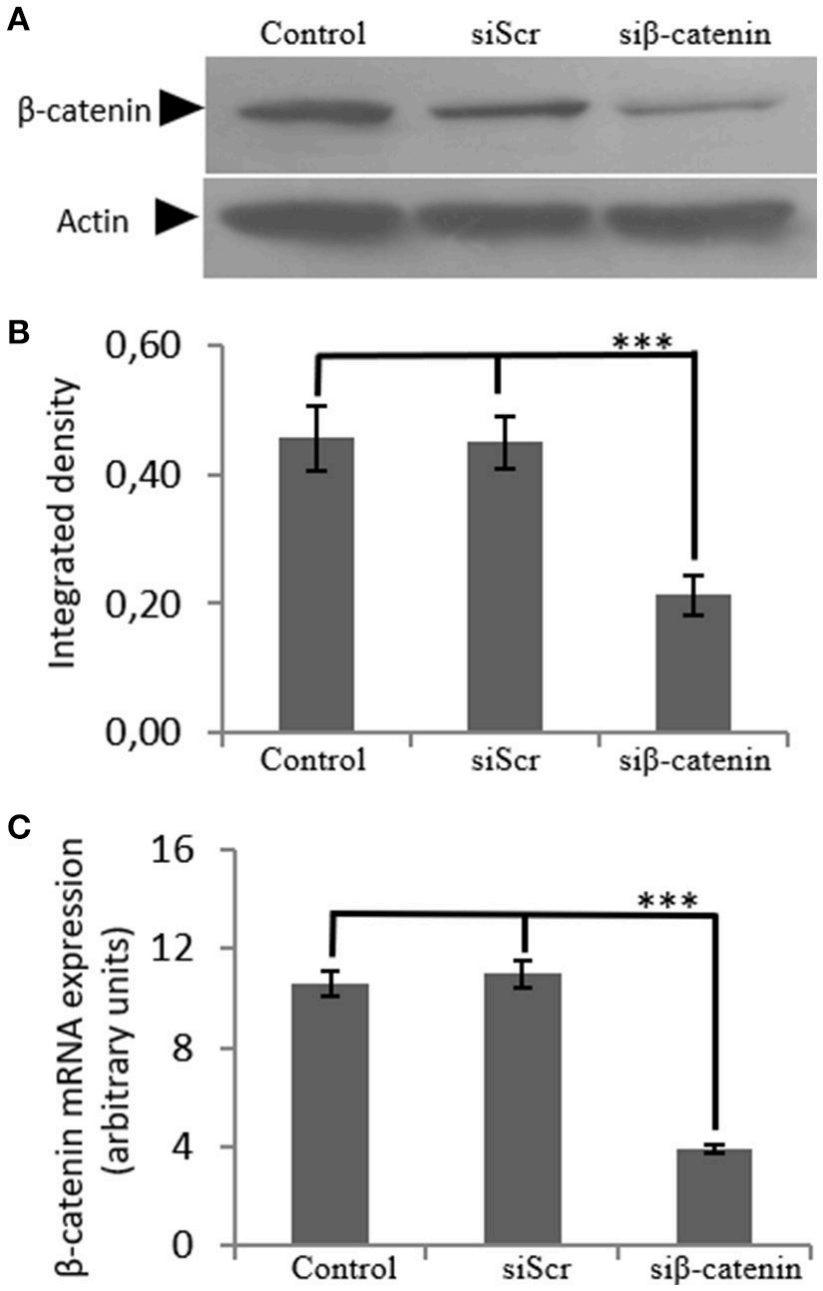
Transfection with siRNA-specific for β-catenin. **(A)** B-catenin protein expression of cells treated with 0% FBS-medium (control) and cells transfected with either siRNAs (short interfering RNAs) against β-catenin (siβ-catenin) or scrambled siRNAs (siScr) were determined by Western blot analysis. **(B)** Densitometric analysis of β-catenin protein levels of the protein band swere normalized against actin and plotted. Representative blots are presented. **(C)** B-catenin mRNA levels in MG63 cells treated with serum-free medium (control) and cells transfected with either siRNAs (short interfering RNAs) against β-catenin (siβ-catenin) or scrambled siRNAs (siScr) were determined by real time PCR using primers specific for the β-catenin gene and normalized against GAPDH. Results represent the average of three separate experiments. Means ± S.E.M were plotted; statistical significance: ^***^p ≤ 0.001 compared with the respective control samples.

In order to investigate the role of β-catenin in the biglycan regulated IGF-IR signaling of MG63 cells, RNA interference methodology was utilized. MG63 cells were transfected with siRNAs against β-catenin (siβ-catenin) for 48 h and mRNA and protein expression of β-catenin were analyzed using Real Time PCR and Western blot analysis, respectively. This strategy resulted in an efficient downregulation of β-catenin at both protein (*p* ≤ 0.001) and mRNA levels (*p* ≤ 0.001; Figure 7).

**Figure 8:**
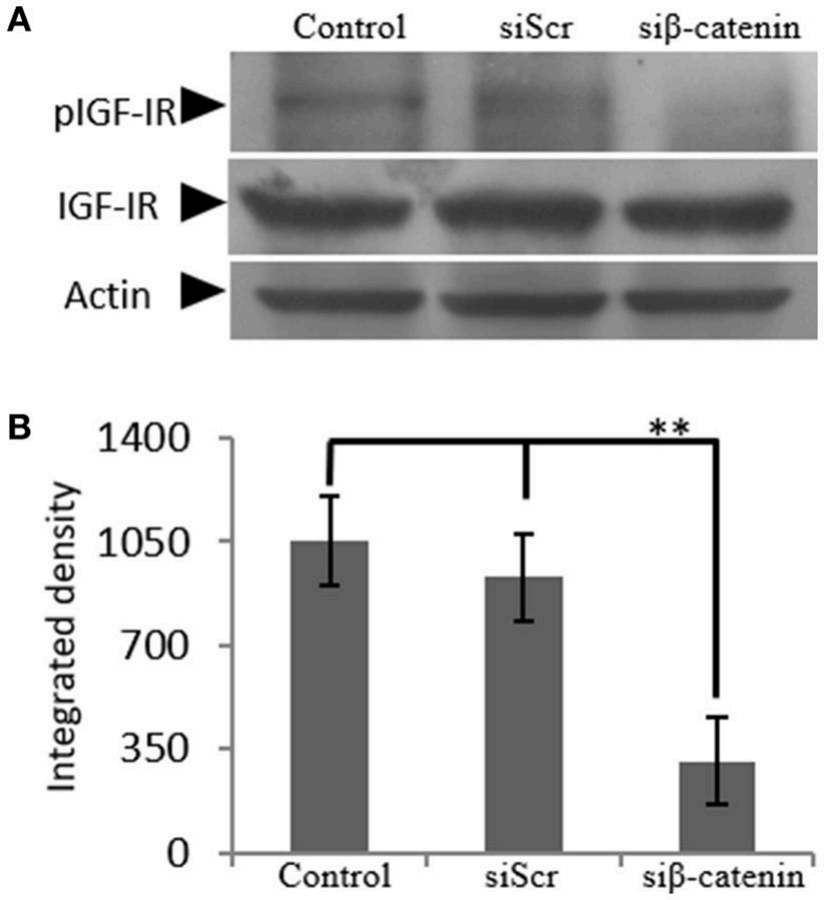
Effect of siβ-catenin on the activation of IGF-IR. **(A)** Expression of IGF-IR total protein (IGF-IR) and phosphorylated IGF-IR protein (pIGF-IR) of cells treated with 0% FBS-medium (control) and cells transfected with either siRNAs (short interfering RNAs) against β-catenin (siβ-catenin) or scrambled siRNAs (siScr) were determined by Western blot analysis. **(B)** Densitometric analysis of the activated IGF-IR levels (pIGF-IR/IGF-IR) from the protein bands were normalized against actin and plotted. Representative blots are presented. Results represent the average of three separate experiments. Means ± S.E.M were plotted; statistical significance: ^**^p ≤ 0.01 compared with the respective control samples.

The generated β-catenin-deficient cells were used to study the possible effect of β-catenin/Wnt pathway on IGF-IR activation. Thus, phoshorylation of IGF-IR was assessed using Western blot analysis of protein extracts from cells incubated with 0% FBS medium (C), cells transfected with siβ-catenin and cells transfected with scrambled siRNAs (siScr). Interestingly, a strong decrease in the phosphorylation levels of IGF-IR (*p* ≤ 0.01) in β-catenin deficient cells was shown (Figure 8) suggesting that β-catenin/Wnt pathway can regulate IGF-IR activation in MG63 osteosarcoma cells.

### Biglycan co-localizes with LRP6

**Figure 9:**
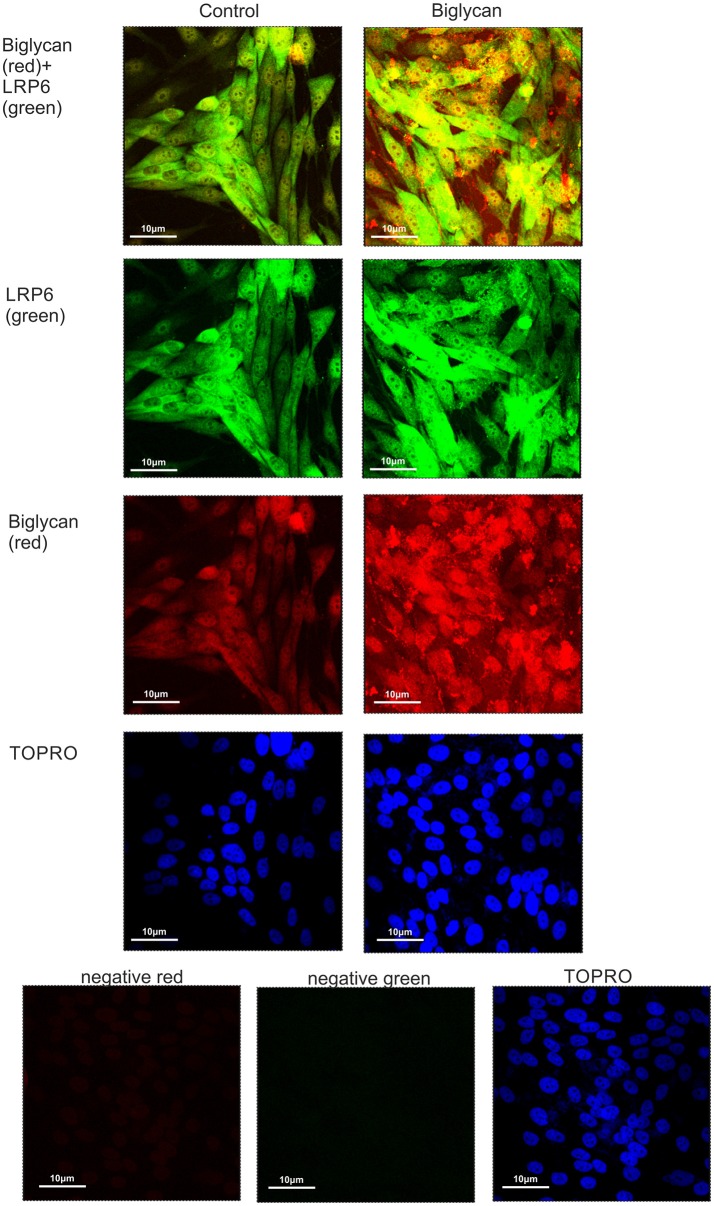
Co-localization of biglycan and LRP6 in MG63 cells using immunofluorescence. Biglycan (red; anti-mouse Alexa Fluor 555) and LRP6 (green; anti-rabbit Alexa Fluor 488) protein staining of cells and respective nuclear staining (using TO-PRO-3) were evaluated in cultures after 48 h in serum-free medium (control) or biglycan (10 μg/ml). In negative controls, primary antibodies were omitted, but both secondary antibodies used (anti-mouse - negative red; anti-rabbit—negative green). Slides were analyzed by confocal microscopy and pictures were taken using x40 magnification.

Previously, biglycan was shown in osteoblasts to affect Wnt-induced β-catenin/T cell-specific factor-mediated transcriptional activity by interacting with the LRP6 receptor (26). Therefore, we examined whether in our osteosarcoma model biglycan interacts with the LRP6 receptor. Utilization of immunofluorescence demonstrated an abundant deposition of LRP6 (green color) and associated to the cell membrane biglycan (red color with moderate co-localization (LRP6 + biglycan; Figure 9). Upon treating the cells with recombinant biglycan a strong increase in orange color, together with enhanced biglycan-LRP6 co-localization was showed (Figure 9). These data demonstrate that biglycan in a concentration dependent manner co-localizes with LRP6 in MG63 osteosarcoma cells. Therefore, biglycan, through its interaction with LRP6, activates the receptor and attenuates β-catenin degradation.

### Cross talk between β-catenin and IGFR signaling

**Figure 10:**
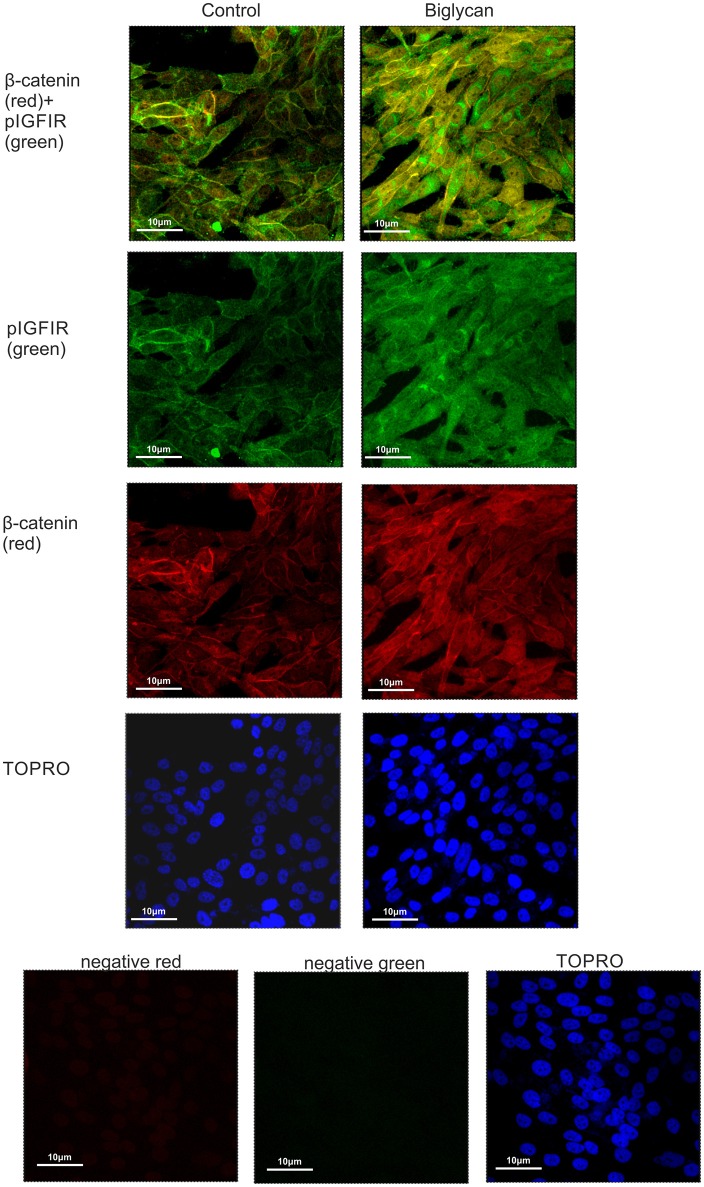
Co-localization of β-catenin and pIGF-IR in MG63 cells using immunofluorescence. B-catenin (red; anti-mouse Alexa Fluor 555) and pIGF-IR (green; anti-rabbit Alexa Fluor 488) protein staining of cells and respective nuclear staining (using TO-PRO-3) were evaluated in cultures after 48 h of incubation with 0% FBS-medium (control) or biglycan (10 μg/ml). Negative controls were used wherethe primary antibodies were omitted for both secondary antibodies used (anti-mouse - negative red; anti-rabbit—negative green). Slides were analyzed by confocal microscopy and pictures were taken using x40 magnification.

Cytoplasmic β-catenin can be complexed to the cell membrane with the cadherin family members (33). We hypothesized that β-catenin can interact with IGF-IR and to facilitate signaling. Applying anti-β-catenin (green color) and anti-pIGF-IR (red color) antibodies to MG-63 cells demonstrated a co-localization between these molecules (Figure 10). Observed co-localization was enhanced in biglycan treated MG-63 cells (Figure 10).

### Biglycan activates canonical β-catenin pathway in MG63 cells

**Figure 11:**
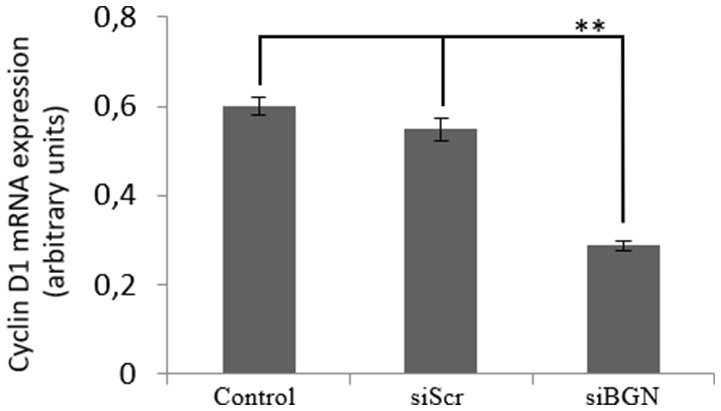
Effect of biglycan in the mRNA expression of cyclin D1. Cyclin D1 mRNA levels in MG63 cells treated with 0% FBS-medium (control) and cells transfected with either siRNAs (short interfering RNAs) against biglycan (siBGN) or scrambled siRNAs (siScr) were determined by real time PCR using primers specific for the biglycan gene and normalized against GAPDH. Results represent the average of three separate experiments. Means ± S.E.M were plotted; statistical significance: ^**^p ≤ 0.01 compared with the respective control samples.

Wnt-mediated transcription through β-catenin—T-cell factor (TCF)/Lymphoid enhancer-binding factor (Lef) transcription factors, is characterized as the canonical wnt/β-catenin signaling pathway. To assess the modulation of this pathway by biglycan we examined the expression of a β-catenin downstream target molecule cyclin D1 (34, 35). As demonstrated in Figure 11 cyclin D1 expression was significantly (*p* ≤ 0.01) downregulated in biglycan-deficient cells, suggesting that biglycan affects both canonical and non-canonical signaling in osteosarcoma cells.

### Role of ERK1/2 in the biglycan regulated IGF signaling

**Figure 12:**
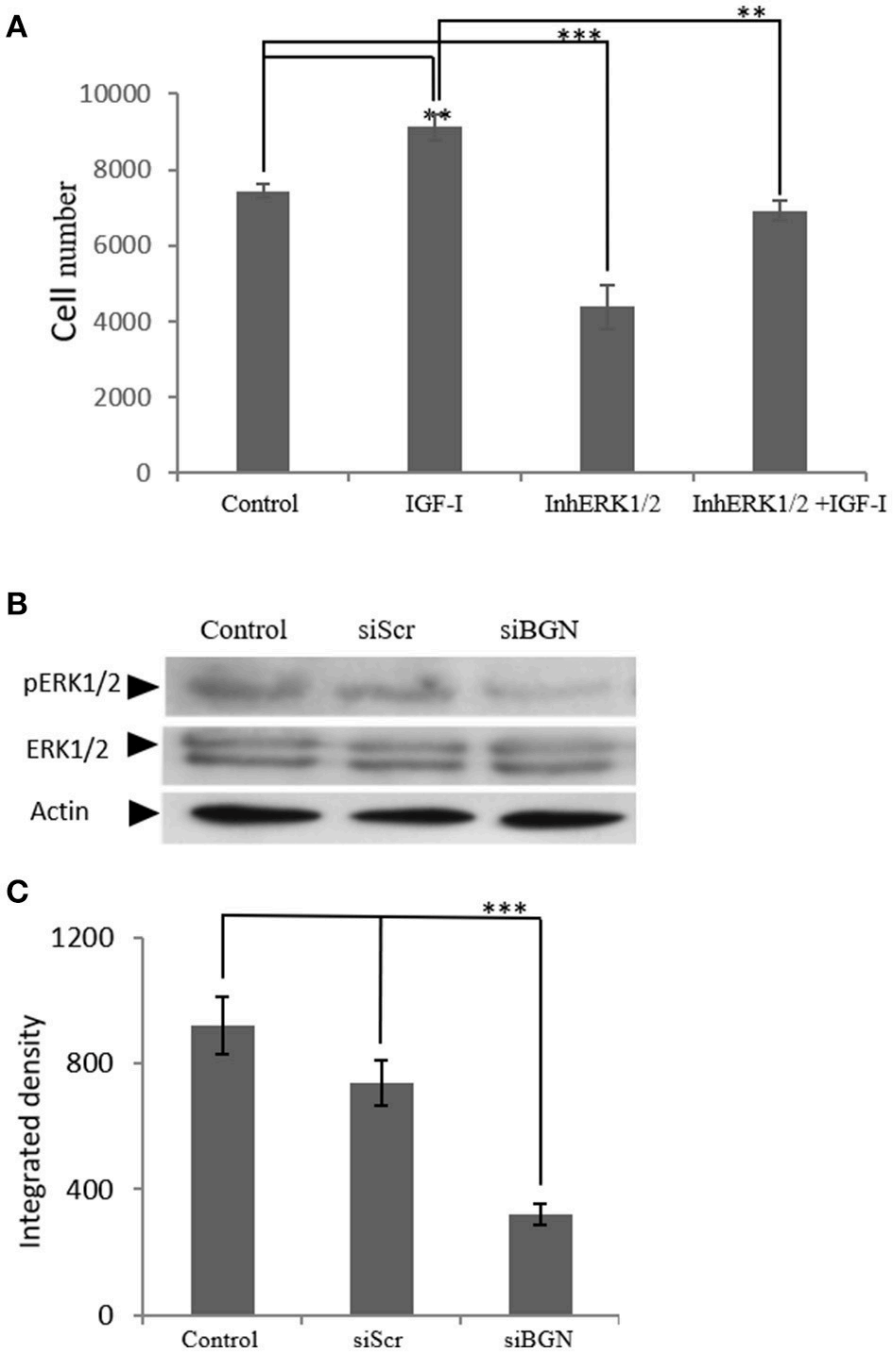
The role ofERK1/2 on MG63 cell proliferation and the effect of biglycan expression on its activation. **(A)** MG63 cells were harvested and seeded (3,500 cells/well) on 96-well plates. Cells, in each well, incubated with serum-free medium (control), 10 ng/ml IGF-I (IGF-I), 5 μM ERK1/2 inhibitor (InhERK1/2), and 10 ng/ml IGF-I + 5 μM ERK1/2 inhibitor (IGF-I+InhERK1/2), were counted using fluorometric CyQUANT assay kit. **(B)** Expression of ERK1/2 total protein (ERK1/2) and phosphorylated ERK1/2 protein (pERK1/2) of cells incubated in serum-free medium (control) and cells transfected with either siRNAs against biglycan (siBGN) or scrambled siRNAs (siScr) were determined by Western blot analysis. **(C)** Densitometric analysis of the activated ERK1/2 levels (pERK1/2 /ERK1/2) from the proteinbandswere normalized against actin and plotted. Results represent the average of three separate experiments. Means ± S.E.M were plotted; statistical significance: ^***^p ≤ 0.001, ^**^p ≤ 0.01 compared with the respective control samples.

IGF-I/IGF-IR are known to activate several downstream signaling pathways that regulate cell functions including that of the MAP kinases (36, 37). Thus, the role of ERK1/2, member of the MAP kinase pathway, was investigated on IGF-I/biglycan growth effects. Utilization of an ERK1/2 inhibitor in MG63 cells treated or not with IGF-I (10 ng/mL) demonstrated a significant decrease in both the basal and the IGF-I induced MG63 cell proliferation (*p* ≤ 0.01 and *p* ≤ 0.001, respectively; Figure 12A). These data, therefore, suggest that ERK participates in the regulation of both basal and IGF-I dependent MG-63 cell growth. Next, we wanted to examine the putative effects of biglycan on ERK1/2 activation. To achieve this aim, analysis of ERK1/2 phosphorylation was performed in cell extracts of control cells, cells transfected with siRNAs against biglycan (siBGN) and cells transfected with negative siRNAs (siScr). As shown in Figures 12 B,C the activation of ERK1/2 was significantly (*p* ≤ 0.001) inhibited in biglycan-deficient (siBGN) cells. These results suggest that ERK1/2 is a downstream mediator of IGF-IR/biglycan signaling dependent cell growth.

## Discussion

Biglycan, an important component of bone ECM, has been correlated to the emergence of mesenchymal tumors (7, 21, 38). Recent publications show the importance of the “proteoglycan signature” on cancer patients' outcome (38) and discuss that they can be used as sensitive biomarkers/therapy targets in some cancer types (39–43). In the present study we demonstrate for the first time that biglycan is an endogenous upregulator of osteoasarcoma growth as biglycan-deficient cells were shown to have attenuated ability to proliferate. Others, upon dissecting the sets of genes that regulate the cell functions of biglycan-null pre-osteoblasts using oligonucleotide microarrays, have previously shown that biglycan deficiency modulates the genes that regulate inflammation processes, immune response, as well as proliferation of tumor cells (44). Furthermore, osteosarcoma markers of poor response to therapy (Huvos grade I/II response defines tumors with little or no response to chemotherapy) are predominantly gene products involved in microenvironmental remodeling and osteoclast differentiation, including biglycan (14). We further investigated if other potential mediators of biglycan action involved in key developmental processes such as BMP, Wnt/β-catenin, RUNX2, HIPPO/YAP or IGF-IR are “hijacked” by osteosarcoma (45) Previously, IGF-I was shown to be an important enhancer of osteosarcoma cell survival facilitating growth and attenuating apoptosis (46). Furthermore, IGF-IR is upregulated in osteosarcoma tissue samples as recently shown by Liu et al. (47) whereas the genetic polymorphisms of IGF-I were shown to be correlated with osteosarcoma risk and prognosis (48). Since in the present study, we showed an enhancement of biglycan expression through IGF-IR downstream effects we examined the possible involvement of IGF-IR in biglycan-dependent osteosarcoma cell growth. Indeed, in this study IGF-IR downstream signaling, was shown to be a strong endogenous enhancer of MG63 cell growth, in keeping with previous reports (47, 49). Importantly, IGF-I/IGF-IR dependent growth was completely abrogated in biglycan-deficient cells. Previously, it has been demonstrated that ligand-induced IGF-IR activation is followed by proteasomal and lysosomal degradation of IGF-IR, a phenomenon of receptor desensitization (50). Thus, endogenous mechanisms prolonging IGF-IR activation would lead to a protracted IGF-I-dependent growth response and facilitate cancer progression. We hypothesized that biglycan downstream effects could lead to enhanced IGF-IR activation and prolonged growth response and assessed potential mediators focusing on the Wnt/β-catenin. The indirect interactions of β-catenin with IGF-IR receptor and other members of the IGF-I signaling pathway have been reported as β-Catenin/POU5F1/SOX2 transcription factor complex mediates IGF-I receptor signaling and predicts poor prognosis in lung adenocarcinoma and Caveolin-1-LRP6 signaling module stimulates aerobic glycolysis in prostate cancer (27–29). On the other hand, it has been shown that biglycan regulates Wnt/β-catenin pathway in osteblastic-lineage cells, by either mediating ligand effects or modulating downstream signaling molecules (26).

We examined the possibility of a cross-talk between IGF-IR and Wnt/β-catenin pathways in the biglycan effect on osteosarcoma growth. Interestingly, in this study β-catenin deficient MG63 osteosarcoma cells were shown to have abrogated both basal and IGF-I-dependent IGF-IR activation, and strongly attenuated growth. Likewise, we demonstrate for the first time that treatment of MG63 osteosarcoma cells with recombinant biglycan increases β-catenin expression and mediates its cellular deposition and enhancingβ-catenins' cell membrane and cytoplasmic localizations. Previously, it has been shown in mouse calvarial osteoblasts model, that biglycan interacts with both the canonical Wnt ligand Wnt3a and the Wnt co-receptor low-density lipoprotein receptor-related protein 6 (LRP6). Furthermore, this co-localization enhanced downstream Wnt-induced β-catenin/T cell-specific factor-mediated transcriptional activity (26). In order to clarify the mechanism of biglycan action we examined biglycan and LRP6 respective localization in MG63 cells utilizing immunofluorescence assays. This approach showed that biglycan co-localizes with LRP6 in a manner dependent on biglycan concentration. Binding of biglycan to (LRP5/6) co-receptors determines the inactivation of the β-catenin destruction complex and increases β-catenin cytoplasmic pool. This allows β-catenin to accumulate in the cytoplasm and translocate to the nucleus, where it forms a transcriptionally active complex with TCF/LEF family members or to engage in interactions with and stabilize its membrane/cytoplasmic pool (51). β-catenin cytoplasmic pool is deregulated in cancer in a manner correlated to cancer development (52).

Activation of Wnt signaling was previously shown to enhance the survival of osteoblastic cells, as demonstrated by the reduction in osteoblast and osteocyte apoptosis in mice not expressing the Wnt inhibitor sFRP1 (soluble frizzled-related protein 1) (53). Moreover, Wnt proteins were demonstrated in a separate study to prevent osteoblast and osteocyte apoptosis by a mechanism that requires activation of the Src/ERK signaling pathway (54). Furthermore, increased insulin-like growth factor receptor (IGF-1R) protein levels and an activation of the phosphatidylinositol 3-kinase/Akt/glycogen synthase kinase 3 beta/β-catenin signaling pathway were identified in a mouse neurodegenerative disease model (55). In the present study a strong up regulation β-catenin to the MG63 cell cytoplasm as well as moderate upregulation to membrane and to nucleus was evidenced upon biglycan treatment. Furthermore, immunofluorescence demonstrated that increased MG63 cell growth correlated with β-catenin colocalizing with IGF-IR to the membrane, cytoplasm and nucleus. Previously in a number of chordoma biopsies, Aleksic et al. detected IGF-1R in the plasma membrane and cytoplasm which was expressed more strongly in recurrent tumor than the primary (56). Furthermore, in the same cohort Aleksic et al. had identified heterogeneous nuclear IGF-1R, which has been linked with sensitivity to IGF-1R inhibition (56).

Our present data demonstrate that biglycan, by binding to the LRP6 receptor, inactivates inactivates the formation of the β-catenin destruction complex enhancing its cytoplasmic, membrane as well as nuclear deposition. Increased β-catenin pool interacts with IGF-IR, strongly enhancing pIGF-IR expression at different subcellular compartments. Furthermore, ERK1/2 was found to be a downstream mediator of biglycan/IGF-IR/β-catenin signaling axis facilitating MG63 cell growth. However, we do not exclude the possibility that ERK1/2 may be a downstream mediator of other membrane receptors including Wnt receptors (57) or that ERK1/2 contributes to the sustaining of the β-catenin cytoplasmic pool. Indeed, other studies have suggested an interaction(s) between the β-catenin/Wnt and the ERK pathway without clarifying the exact mechanism(s) of action (58–60). It has, however, been established in NIH 3T3 cells, that WNT3A induces cell proliferation through the activation of ERK and Wnt/β-catenin pathways indicating that these two signaling cascades interact at several levels (61). Moreover, in this study, we also confirm that an increased β-catenin nuclear translocation facilitated transcription of target cyclin D1 gene involved in cell cycle regulation, as previously well established in other models (62).

Our study presents some limitations. First, we utilized a single cell line (MG63), and second, we have not identified the components of the IGF-IR/β-catenin co-localization complex, an issue currently under investigation. Future studies in different *in vitro/in vivo* models as well as interpolation with data obtained from patient biopsies could provide more refined data to the mechanism presented here.

**Figure 13:**
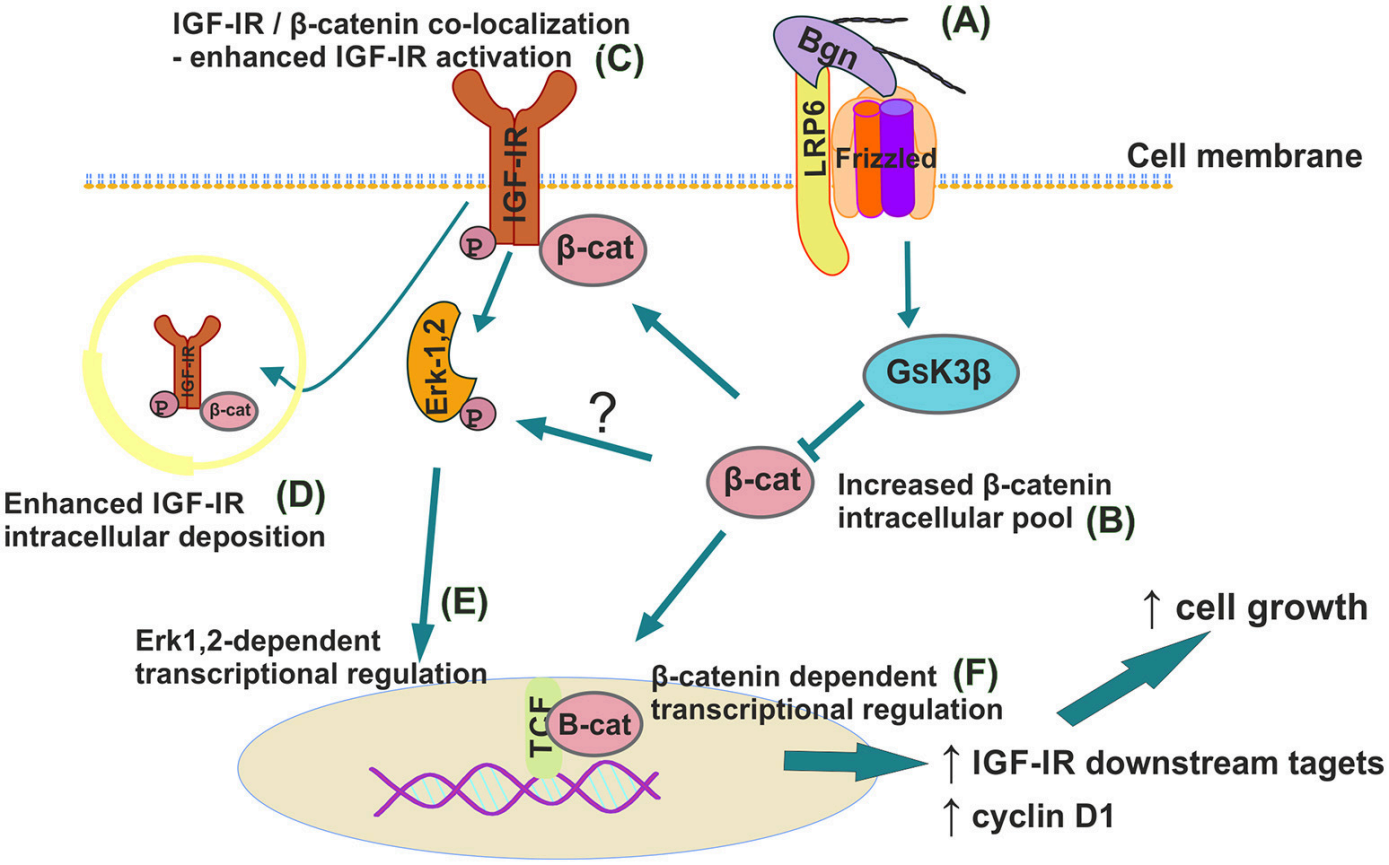
Schematic presentation of proposed biglycan/LRP6/IGF-IR downstream signaling axis in regulation of MG63 osteosarcoma cell growth. **(A)** Secreted to ECM biglycan binds to LRP6 and activates frizzled to de-activate GSK3β. **(B)** β-catenin cytoplasmic pool increases. **(C)** β-catenin co-localizes with IGF-IR to enhance its activation/deposition to membrane and to **(D)** cytoplasm. **(E)** pIGF-IR activates ERK1,2 to induce downstream transcriptional regulation. **(F)** Part of cytoplasmic β-catenin pool translocates to nucleus to induce transcriptional regulation of target genes.

In summary we report a novel mechanism where biglycan, an ECM proteoglycan, through a LRP6/β-catenin/IGF-IR axis enhances osteosarcoma cell growth (Figure 13). IGF-IR activation results in increased biglycan secretion thus, forming an autonomous ECM-originating signaling loop which contributes to osteosarcoma growth. Previously PGs were discussed to “regulate the bioavailability of hormones, growth factors, and cytokines as well as the activation of their respective receptors which regulate phenotypic diversibility, gene expression and rates of recurrence in specific tumor types” (40). Defining and targeting the components of the biglycan signaling loop on an individual patient may basis offer ground for the generation of tailor-made osteosarcoma strategies?

## Author contributions

JA, AB, DN, DP, AT, and GT conceived and designed the experiments and wrote the paper. JA, AB, DN, DP, AP, and AT performed the experiments and analyzed the data. All authors approved the manuscript.

### Conflict of interest statement

The authors declare that the research was conducted in the absence of any commercial or financial relationships that could be construed as a potential conflict of interest.
