# Supplementary figures and images for: Biglycan Regulates MG63 Osteosarcoma Cell Growth Through a LPR6/β-Catenin/IGFR-IR Signaling Axis
Source: Front Oncol. 2018 Oct 23;8:470. doi: 10.3389/fonc.2018.00470 (PMC6206209; doi:10.3389/fonc.2018.00470)

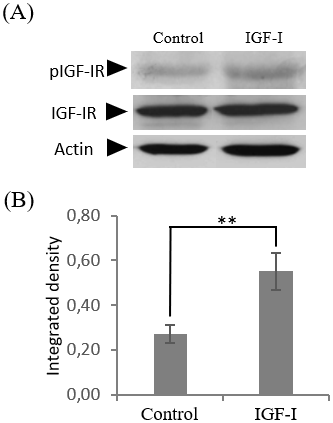

Supplement: Supplementary file 1 [file Image_1.TIF]
